# Membrane estrogen receptor-α contributes to female protection against high-fat diet-induced metabolic disorders

**DOI:** 10.3389/fendo.2023.1215947

**Published:** 2023-07-17

**Authors:** Aurélie Fabre, Blandine Tramunt, Alexandra Montagner, Céline Mouly, Elodie Riant, Marie-Lou Calmy, Marine Adlanmerini, Coralie Fontaine, Rémy Burcelin, Françoise Lenfant, Jean-François Arnal, Pierre Gourdy

**Affiliations:** ^1^ Institut des Maladies Métaboliques et Cardiovasculaires (I2MC), Institut National de la Santé et de la Recherche Médicale (INSERM)/Université Paul Sabatier (UPS), Université Toulouse 3, Toulouse, France; ^2^ Service de Diabétologie, Maladies Métaboliques et Nutrition, Centre Hospitalier Universitaire (CHU) de Toulouse, Toulouse, France; ^3^ Service d’Endocrinologie et Nutrition, Centre Hospitalier Universitaire (CHU) de Toulouse, Toulouse, France

**Keywords:** sex differences, estrogen receptor alpha (ERα), membrane-initiated steroid signaling, obesity, insulin resistance, thermogenesis

## Abstract

**Background:**

Estrogen Receptor α (ERα) is a significant modulator of energy balance and lipid/glucose metabolisms. Beyond the classical nuclear actions of the receptor, rapid activation of intracellular signaling pathways is mediated by a sub-fraction of ERα localized to the plasma membrane, known as Membrane Initiated Steroid Signaling (MISS). However, whether membrane ERα is involved in the protective metabolic actions of endogenous estrogens in conditions of nutritional challenge, and thus contributes to sex differences in the susceptibility to metabolic diseases, remains to be clarified.

**Methods:**

Male and female *C451A-ERα* mice, harboring a point mutation which results in the abolition of membrane localization and MISS-related effects of the receptor, and their wild-type littermates (*WT-ERα*) were maintained on a normal chow diet (NCD) or fed a high-fat diet (HFD). Body weight gain, body composition and glucose tolerance were monitored. Insulin sensitivity and energy balance regulation were further investigated in HFD-fed female mice.

**Results:**

*C451A-ERα* genotype had no influence on body weight gain, adipose tissue accumulation and glucose tolerance in NCD-fed mice of both sexes followed up to 7 months of age, nor male mice fed a HFD for 12 weeks. In contrast, compared to WT-ERα littermates, HFD-fed *C451A-ERα* female mice exhibited: 1) accelerated fat mass accumulation, liver steatosis and impaired glucose tolerance; 2) whole-body insulin resistance, assessed by hyperinsulinemic-euglycemic clamps, and altered insulin-induced signaling in skeletal muscle and liver; 3) significant decrease in energy expenditure associated with histological and functional abnormalities of brown adipose tissue and a defect in thermogenesis regulation in response to cold exposure.

**Conclusion:**

Besides the well-characterized role of ERα nuclear actions, membrane-initiated ERα extra-nuclear signaling contributes to female, but not to male, protection against HFD-induced obesity and associated metabolic disorders in mouse.

## 1 Introduction

Besides their pivotal role in sexual development and reproduction, estrogens, the major female sex steroid hormones have been recognized as key players in the preservation of glucose homeostasis and energy balance (1, 2). Therefore, they contribute to sex differences in the susceptibility to metabolic diseases such as type 2 diabetes (T2D) or non-alcoholic fatty liver diseases (NAFLD) (2, 3). Accordingly, both clinical observations and experimental studies demonstrated that deficient endogenous estrogen production, as observed after menopause in women or bilateral oophorectomy in animal models, promotes fat mass accumulation, insulin resistance and hyperglycemia, unfavorable features that can be prevented by chronic administration of 17β-estradiol (E2) (2, 3).

Estrogen signaling mainly involves two members of the nuclear receptor superfamily, namely the estrogen receptor alpha (ERα, *Esr1* gene) and the estrogen receptor beta (ERβ, *Esr2* gene) (4). In the past few decades, it has become apparent that ERα mediates the vast majority of metabolic effects of estrogens. Indeed, unlike the results reported in ERβ-deficient (*ERβ^-/-^*) mice, both male and female *ERα^-/-^* mice demonstrated massive adiposity, insulin resistance, and impaired glucose tolerance as compared to their wild-type littermates (5–7), in line with the phenotype observed in rare individuals carrying inactivating mutations of the receptor (8). Moreover, E2 administration failed to prevent high-fat diet (HFD)-induced obesity and metabolic disorders in *ERα^-/-^* (but not *ERβ^-/-^*) oophorectomized female mice (7, 9).

ERα signaling has been primarily described at the nuclear level where the receptor acts as a modulator of transcription through direct or indirect interaction with target genes (4, 10). Recent studies using mouse models abrogating the nuclear actions of ERα provided evidence that this classical pathway plays a major role in metabolic protection conferred by estrogens (11). The prevention of HFD-induced obesity and insulin resistance by either endogenous estrogens or E2 administration is thus totally abolished in mice selectively deficient in ERαAF-2, an activation function which is critical for the transcriptional activation of ERα by E2 through the recruitment of coactivators (7). Furthermore, AF1 activation by E2 requires functional AF2, and *ERαAF-2°* mice can thus be considered as a model deficient in nuclear ERα actions (10, 12).

In addition to this classical nuclear function of ERα, further experimental evidence, coming first from *in vitro* studies, showed that ERα is also able to mediate extranuclear signaling. Indeed, rapid signaling elicited by a subcellular pool of ERα located at the plasma membrane has been characterized in a variety of cell lines and was referred as Membrane-Initiated Steroid Signaling (MISS) (10, 13). In the last years, the development of selective pharmacological tools that specifically activate MISS as well as the generation of mice expressing an ERα protein impeded for membrane localization began to unravel the role of MISS *in vivo* (12, 13). Taking advantage of these original experimental approaches, recent studies suggested that ERα membrane signaling could also contribute, at least in part, to the effects of estrogens on energy balance and glucose metabolism. First, the pharmacological selective activation of membrane ERα by pathway preferential estrogen-1 (PaPE-1) was shown to confer metabolic protection as illustrated by the prevention of body weight gain and fat accumulation in oophorectomized mice (14), while estrogen dendrimer conjugates (EDC) protected against western diet (WD)-induced liver steatosis (15) and enhanced pancreatic β-cell function and survival (16, 17). Then, the study of a new mouse model carrying a mutation in the palmitoylation site of ERα (*C451A-ERα*) required for membrane localization of the receptor (*C451A-ERα* mouse, also referred as Nuclear-Only Estrogen Receptor *(NOER)* mouse by E. Levin’s group) indicated that MISS effects could participate to suppression of adipogenesis (18), and that *NOER* females but not males fed a chow diet (CD) exhibit mild hepatic insulin resistance and glucose intolerance (19).

Altogether, recent data thus suggest the contribution of ERα-MISS effects in the regulation of energy balance and metabolism, besides the well-characterized role of ERα nuclear signaling. However, whether membrane ERα is involved in the protection conferred by endogenous estrogens in conditions of nutritional challenge and thus contributes to sex differences in the susceptibility to metabolic diseases remains to be clarified. The present study thus investigated the influence of HFD feeding in *C451A-ERα* mice, demonstrating sex-specific exacerbation of fat mass accumulation and metabolic disorders in females, associated with impairment in energy expenditure.

## 2 Materials and methods

### 2.1 Animals

The *C451A-ERα* knock-in mouse line was generated on a C57Bl6/N background as previously described (20). Wild-type littermates (*WT-ERα*) were systematically used as controls. Animals were housed in groups of 6 and kept in a specific pathogen-free and temperature-controlled facility on a 12-h light/dark cycle. All experimental procedures were performed in accordance with the principles established by EU directive 2010/63/EU, the Institut National de la Santé et de la Recherche Médicale and approved by the local Ethical Committee of Animal Care (CEA-122-DAP-2014-56).

Five-week-old mice were fed with either a normal chow diet (NCD; 2.9 kcal/g; SAFE, Augy, France) or a high-fat diet (HFD; 45% fat, 3.7 kcal/g; Research Diets, New Brunswick, NJ). Food intake and body weight were recorded weekly. Body composition determination by EchoMRI, assessment of basal metabolism using metabolic cages, intraperitoneal glucose tolerance tests (IPGTT) and hyperinsulinemic euglycemic clamps were performed as previously described (9). Before sacrifice, animals were anesthetized using a combination of 100 mg/kg ketamine hydrochloride (Merial, Lyon, France) and 5 mg/kg xylazine (Sigma-Aldrich, Isle d’Abeau Chesnes, France) administered by intra-peritoneal injection. Following cervical dislocation, organs were removed, weighted, dissected and used for histological analysis or snap frozen in liquid nitrogen and stored at -80°C.

### 2.2 Adipose tissue and liver histology

Paraffin-embedded 70% ethanol-fixed adipose tissue sections were stained with hematoxylin-eosin (HE). All sections were scanned with a nanozoomer scanner (Hamamatsu Photonics, Hamamatsu, Japan). The number and mean size of adipocytes were estimated by using the ImageJ quantification software. Liver tissues were quickly excised and immediately fixed in 10% formalin for 24 hours. These tissue samples were processed using an automatic-tissue processing machine and followed by embedding in paraffin wax. Thin sections (5 *μ*m) were obtained and stained with hematoxylin-eosin (HE). Additional fresh liver samples were immersed in Tissue-Tek-OCT compound (Sakura, Japan), then frozen in isopentane cooled by liquid nitrogen and cryosections (7µm) were stained with Oil red O to assess neutral lipid accumulation. All sections were scanned with a nanozoomer scanner (Hamamatsu Photonics, Hamamatsu, Japan) and analysed with NDP view software (Hamamatsu Photonics, Hamamatsu, Japan).

### 2.3 Liver lipid analysis

Following homogenization of liver samples in methanol/5mM EGTA (2:1 v/v), lipids corresponding to an equivalent of 1 mg tissue were extracted in chloroform/methanol/water (2.5:2.5:2.1, v/v/v), in the presence of the internal standard: 3 μg of stigmasterol, 3 μg of cholesteryl heptadecanoate and 6 μg of glyceryltriheptadecanoate. Neutral lipids were analyzed by gas-liquid chromatography on a FOCUS Thermo Electron system using a Zebron-1 Phenomenex fused silica capillary columns (5 m x 0.32 mm i.d., 0.50 mm film thickness). Oven temperature was programmed to ramp from 200°C to 350°C, at 5°C per min, and the carrier gas was hydrogen (0.5 bar). The injector was held at 315°C and the detector at 345°C.

### 2.4 Biochemical analysis

Plasma levels of insulin and adipokines were determined using the Multiplex Immunoassay Technology Xmap (MADKMAG-71K-05 and MADPNMAG-70K-01, Milliplex; Millipore, Saint-Quentin-en-Yveline, France). Homeostasis model assessment of insulin resistance (HOMA-IR) was calculated as follows: (fasted insulin [mU/L] x fasted glycemia [mg/dL])/405.

Estradiol, testosterone, progesterone and androstenedione serum concentrations were quantified using a high-sensitive gas chromatography-tandem mass spectroscopy method, as described previously (21).

### 2.5 Exploration of insulin signaling *in vivo*

After 6 weeks of HFD feeding, 12-week-old female mice were fasted for 12h and then received a single intra-peritoneal insulin injection (2 U/kg diluted in PBS). The same volume of vehicle (PBS) was injected in control mice. Fifteen minutes after insulin injection, mice were killed by cervical dislocation, then liver and vastus lateralis (VL) skeletal muscle were rapidly removed, immediately frozen in liquid nitrogen, and stored at -80°C for further analysis.

### 2.6 Western blot analysis

Using a Precellys tissue homogenizer, frozen tissues were homogenized in an ice-cold lysis buffer (150mM NaCl, 50mM TrisHCl pH 7.5, 1% NP40, 1mM EDTA, 0.1% SDS) supplemented with 1mM orthovanadate, 5mM NaF, 0.5mM DTT and a cocktail of protease inhibitors (Complete EDTA-free, Roche), then sonicated and centrifuged at 13,000g for 10 minutes at 4°C. Total protein content was measured using DC protein assay kit (Biorad). Samples containing 60µg total proteins were separated on a 10% SDS/PAGE gel and transferred to a nitrocellulose membrane. The primary antibodies used were directed against phosphoSer473-Akt, Akt (Cell Signaling Technology, #4060 and #4691, respectively) or Gapdh (Santa Cruz Biotechnology, sc-32233). Revelation was performed using an HRP-conjugated secondary antibody and visualized by ECL detection according to the manufacturer’s instructions (Amersham Biosciences/GE Healthcare), using ChemiDoc Imaging System (Bio-RAD). Finally, the densitometric analyses of the bands were performed using the ImageJ software.

### 2.7 RNA preparation and real-time quantitative PCR

Total RNA was prepared using GenElute Mammalian Total RNA Miniprep Kit (Sigma-Aldrich) after tissue homogenization using a Precellys tissue homogenizer (Bertin Technology). One µg total RNA was reverse-transcribed using a High Capacity cDNA Reverse Transcription Kit (Applied Biosystems). The relative gene expression was determined by qPCR performed on ABI StepOnePlus (Life technologies). qPCR data were normalized to HPRT and 36B4 mRNA levels for white adipose tissue (WAT), Ppia mRNA levels for muscle and RPL19 mRNA levels for brown adipose tissue (BAT). Primer sequences are given in **Supplementary Table 1**.

### 2.8 Cold tolerance test

After 12 weeks of HFD feeding at room temperature (25°C), female mice were exposed to a 5-hour period at 4°C (acute cold exposure). Mouse core body temperature was measured using a rectal temperature probe at time 0 (before cold exposure), then every hour during the 5 hours of cold exposure.

### 2.9 Statistical analysis

Results are expressed as mean ± SEM. Analyses were performed using GraphPad Prism 5 software (GraphPad Software, San Diego, CA, www.graphpad.com). To test the effect of *C451A-ERα versus WT-ERα* genotype in each sex, unpaired Student’s t test was performed with Welch’s correction if necessary (variance not equal). Two-way ANOVA analyses were used to assess the interaction between the genotype (*WT-ERα versus C451A-ERα*) and the day period (day *versus* night) regarding the measurements carried out in metabolic cages. In the absence of significant interaction, statistical analyses regarding the respective influence of genotype and day period are provided. In case of significant interaction, Bonferroni post-tests were subsequently performed to compare the different groups two by two. Repeated measures ANOVA were used to compare changes over time between the two genotypes. A value of p<0.05 was considered statistically significant.

## 3 Results

### 3.1 Membrane ERα contributes to protection of female mice against HFD-induced obesity and glucose intolerance

**Figure 1:**
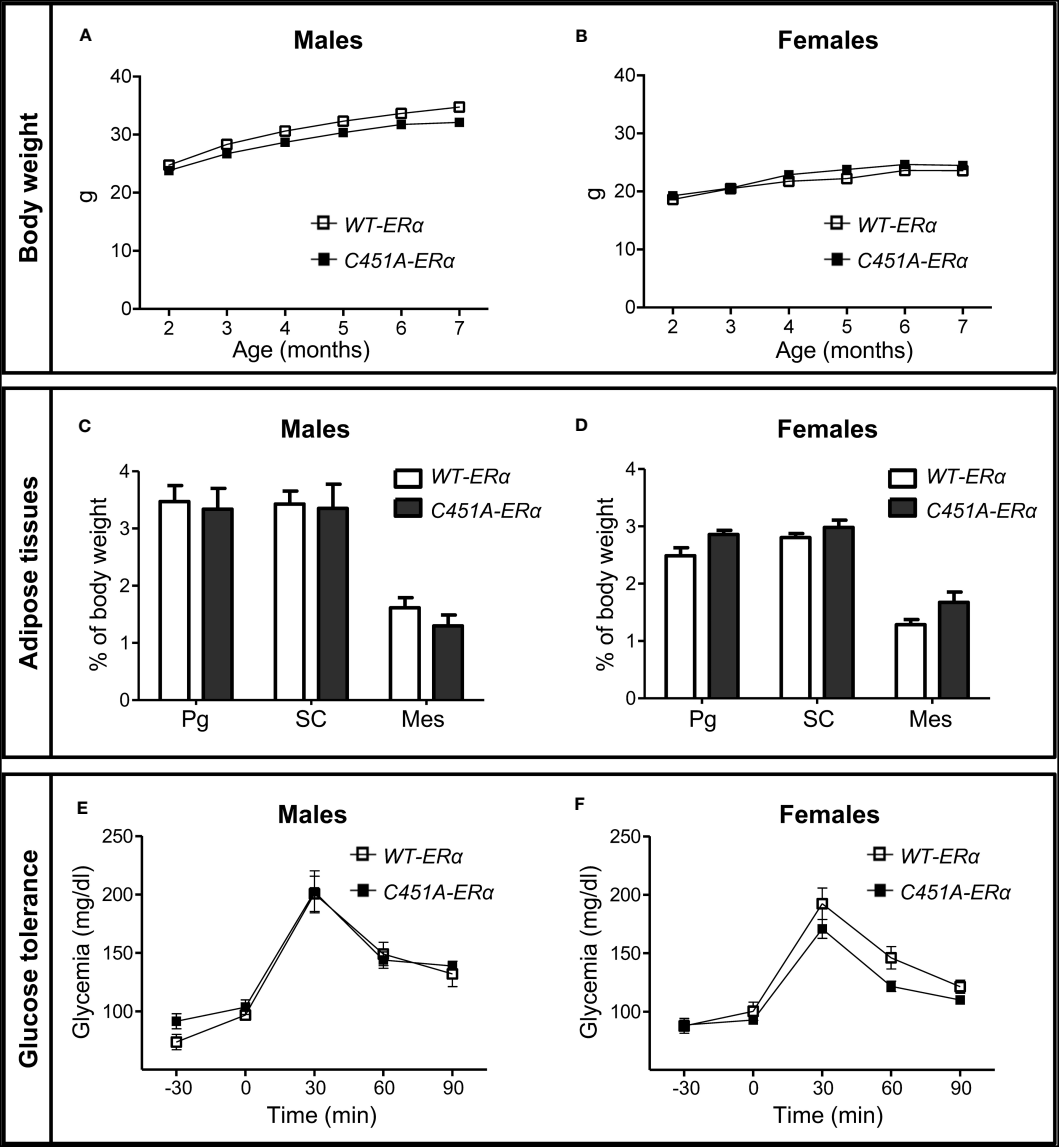
Abrogation of membrane ERα does not influence body weight, adiposity and glucose tolerance in NCD-fed mice. Five-week-old *WT-ERα* and *C451A-ERα* male and female mice were maintained on a normal chow diet (NCD) up to 7 months of age. Body weight **(A, B)**, perigonadal (Pg), sub-cutaneous (SC) and mesenteric (Mes) relative fat pad weights **(C, D)** and intraperitoneal glucose tolerance tests (E and F) are shown in male and female mice, respectively. Data are shown as mean ± SEM (n= 4-14 mice/genotype/sex). **(A-D)** Unpaired Student’s t test was performed. **(E, F)** Repeated measures ANOVA were used to compare changes over time between the two genotypes.

**Figure 2:**
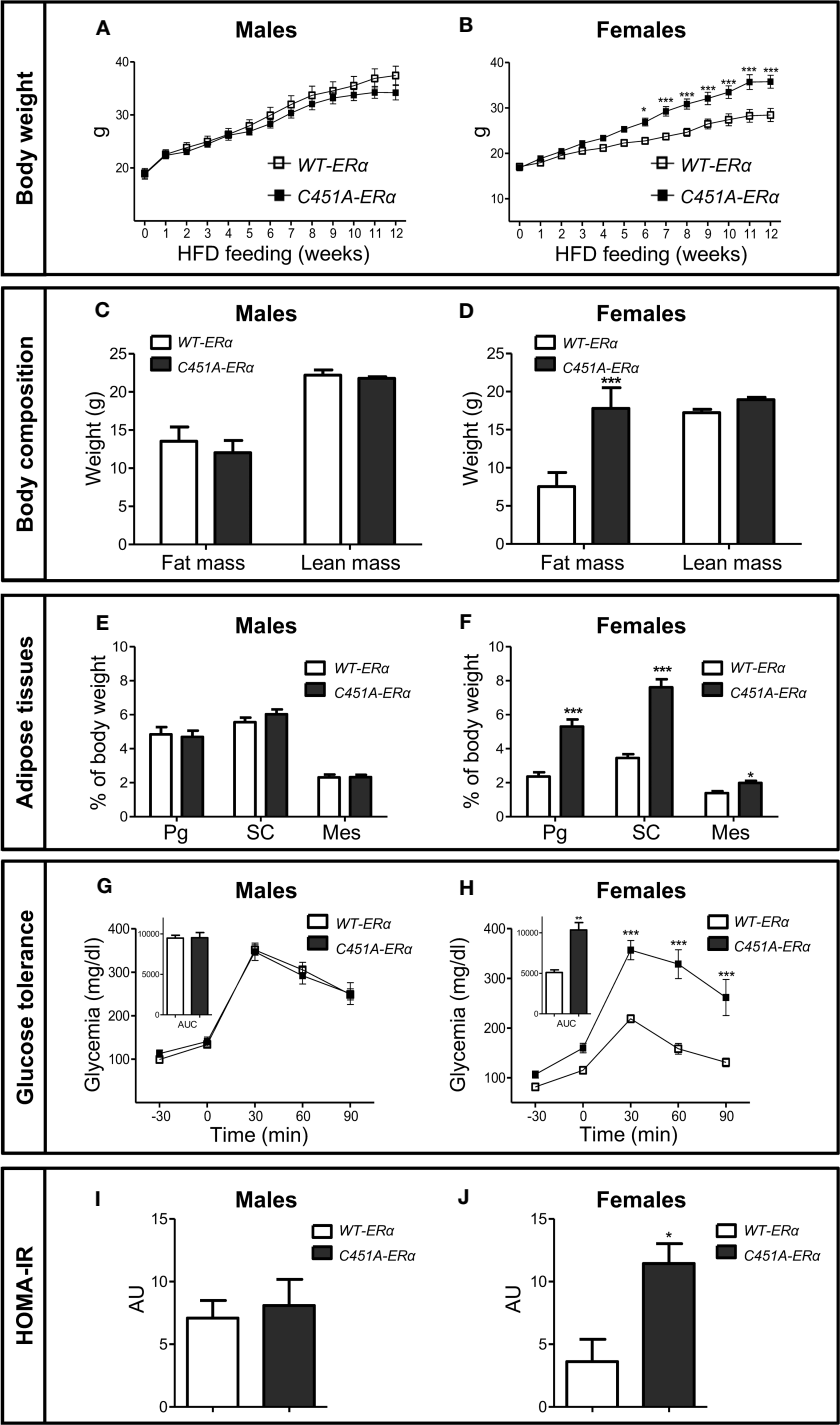
*C451A-ERα* female mice are prone to HFD-induced obesity. Five-week-old *WT-ERα* and *C451A-ERα* male and female mice were fed a HFD for 12 weeks. Body weight **(A, B)**, body composition assessed by EchoMRI **(C, D)**, perigonadal (Pg), sub-cutaneous (SC) and mesenteric (Mes) relative fat pad weights **(E, F)**. Intraperitoneal glucose tolerance test **(G, H)** and HOMA-IR values **(I, J)** are shown in male and female mice, respectively. AUC: Area under the curve. Data are shown as mean ± SEM (n= 10-15 mice/genotype/sex). **(A-F, I, J)** Unpaired Student’s t test was performed. **(G, H)** Repeated measures ANOVA were used to compare changes over time between the two genotypes. For AUC in **(G, H)**, unpaired Student’s t test was performed. *, genotype effect: * p < 0.05, *** p < 0.001.

To explore the influence of ERα-MISS on body weight, body composition and glucose homeostasis, female and male *C451A-ERα* mice and their wild-type littermates (*WT-ERα*) were maintained on a NCD or submitted to a HFD (45% fat). In mice fed the NCD up to 7 months of age, no difference was observed between the two genotypes in terms of body weight gain (**Figures 1A, 1B**), fat mass accumulation in different white adipose tissues (WAT) (**Figures 1C, D**) and glucose tolerance (**Figures 1E, F**), regardless of sex. After 12 weeks of HFD feeding, male *C451A-ERα* mice also exhibited similar body weight gain (**Figure 2A**), body composition (**Figures 2C, E**), glucose tolerance (**Figure 2G**) and insulin sensitivity assessed by HOMA-IR (**Figure 2I**), as compared to *WT-ERα* males. In contrast, HFD-fed female *C451A-ERα* mice were characterized by accelerated body weight gain (**Figure 2B**) and increased fat mass (**Figure 2D**) due to accumulation of adipose tissue in perigonadal and sub-cutaneous fat pads (**Figure 2F**). They also developed more severe glucose intolerance (**Figure 2H**), from the second month of exposure to HFD (**Supplementary Figure 1**), and higher level of HOMA-IR (**Figure 2J**) than their control littermates. Of note, *C451A-ERα* genotype had no influence on daily food consumption in females (9.66 ± 0.27 *versus* 10.51 ± 0.33 kcal/mouse/day in *C451A-ERα* and *WT-ERα* mice, respectively), while a weak but significant decrease in food intake was found in both NCD- and HFD-fed *C451A-ERα* males (**Supplementary Figure 2**). Altogether, these results thus demonstrate that membrane-initiated ERα signaling is involved in the prevention of HFD-induced obesity and hyperglycemia, specifically in female mice.

### 3.2 Loss of ERα-MISS impacts adipocyte and liver adaptation to HFD feeding in female mice

**Figure 3:**
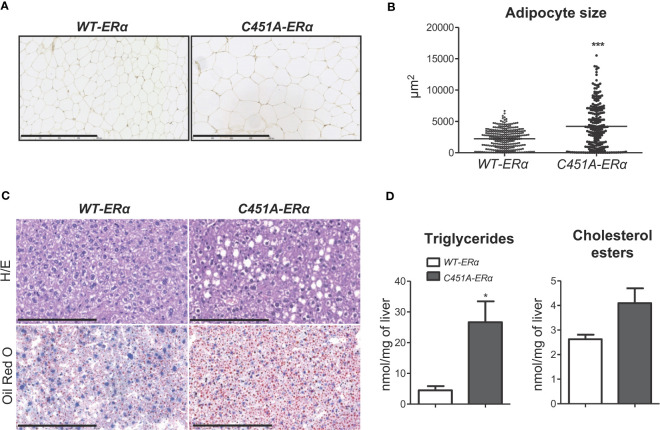
Abrogation of membrane ERα induces adiposity and liver steatosis in HFD-fed female mice. Five-week-old *WT-ERα* and *C451A-ERα* male and female mice were fed a HFD for 12 weeks. Representative pictures (scale bar: 100µm) of perigonadal adipose tissue sections stained with hematoxylin-eosin (H/E) **(A)**, quantification of adipocyte area **(B)**, representative pictures (scale bar: 100µm) of liver histology stained with H/E (top) or oil red O (bottom) **(C)** and relative quantification of triglycerides and cholesterol esters liver content **(D)** are shown. Data are shown as mean ± SEM (n= 4-6 mice/genotype). **(A-D)** Unpaired Student’s t test was performed. *, genotype effect: * p < 0.05, *** p < 0.001.

To further characterize the consequences of membrane ERα defect in HFD-fed female mice, histological analysis of perigonadal WAT was performed, demonstrating a significant increase in mean adipocyte size, with the appearance of very large-sized adipocytes, in *C451A-ERα* as compared to *WT-ERα* mice (**Figures 3A, B**). Liver histology also showed enhanced accumulation of lipid droplets in hepatocytes from HDF-fed *C451A-ERα* female mice, in line with a 4-fold increase in hepatic triglyceride content compared to control littermates (**Figures 3C, D**). Fasting plasma measurements also illustrated the impact of the absence of ERα-MISS on adipocyte and liver biology (**Supplementary Table 2**). Among main adipokines, leptin and resistin concentrations were significantly increased in *C451A-ERα* females, whereas no difference was observed in plasma adiponectin level. In addition, plasma lipid profile of female *C451A-ERα* mice revealed higher levels of total and HDL cholesterol than those found in *WT-ERα* mice. Suggesting more marked hepatic damage, plasma ALT activity levels showed an upward non-significant trend (p=0.06) in *C451A-ERα* female mice.

Plasma samples were also used to determine the influence of ERα-MISS defect on circulating levels of endogenous sex steroids in HFD feeding condition. As previously reported in NCD-fed female *C451A-ERα* mice (20), similar concentrations of E2 were found in both genotypes while non-significant increase in testosterone and decrease in progesterone levels were respectively observed in *C451A-ERα* females compared to control mice (**Supplementary Table 2**).

Altogether, these data indicate that ERα-MISS activity protects against HFD-induced adipocyte hypertrophy and liver steatosis, two hallmarks of impaired lipid metabolism.

### 3.3 ERα-MISS prevents female mice from HFD-induced insulin resistance

**Figure 4:**
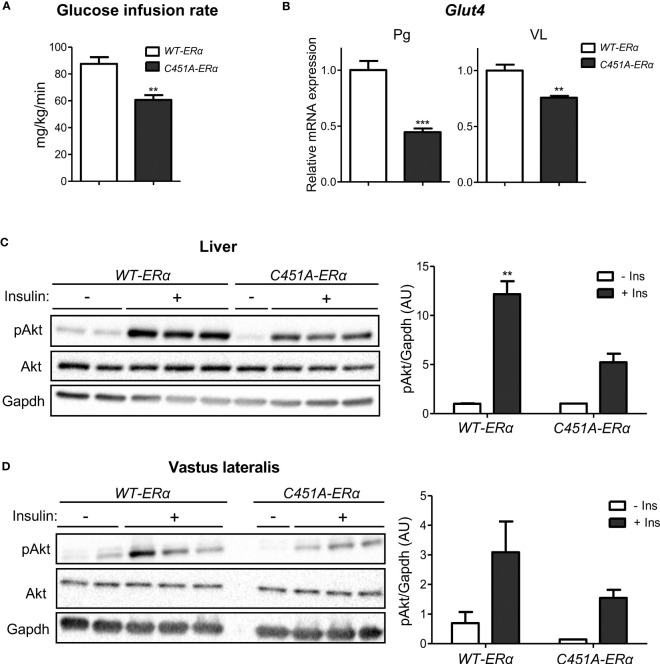
*C451A-ERα* female mice are more susceptible to HFD-induced insulin resistance. Five-week-old *WT-ERα* and *C451A-ERα* male and female mice were fed a HFD for 12 weeks, then submitted to a hyperinsulinemic-euglycemic clamp procedure. Glucose infusion rate **(A)** and relative mRNA expression of *Glut4* in perigonadal adipose tissue (Pg) and muscle (VL, vastus lateralis) at the end of the clamp **(B)** are shown. qPCR data were normalized to HPRT and 36B4 mRNA levels for adipose tissue and to Ppia mRNA levels for muscle. In another experimental set, 8-week-old *WT-ERα* and *C451A-ERα* female mice were fed a HFD for 6 weeks, then administered with a single insulin intra-peritoneal injection (2U/kg diluted in PBS) or vehicle (PBS alone) after a 12h fasting period and sacrificed 15 minutes later. Western blot analysis of Akt phosphorylation on Ser473 in liver **(C)** and muscle (VL, vastus lateralis) **(D)** samples are shown. Gapdh is used as loading controls. Data are shown as mean ± SEM (n= 4-9/genotype). **(A-D)** Unpaired Student’s t test was performed. *, genotype effect: ** p <0.01, *** p <0.001.

Analysis of HOMA-IR levels suggested that female *C451A-ERα* mice were more susceptible to the development of insulin resistance than their *WT-ERα* littermates in response to HFD feeding (**Figure 2J**). The alteration of whole-body insulin sensitivity in *C451A-ERα* females was definitely assessed by hyperinsulinemic-euglycemic clamps, which revealed a 30% decrease in glucose infusion rate as compared to control mice (**Figure 4A**). Accordingly, mRNA expression of the insulin-dependent glucose transporter *Glut4* was significantly decreased in WAT (Perigonadal, Pg) and skeletal muscle (Vastus lateralis, VL) of HFD-fed female *C451A-ERα* mice at the end of the clamp procedure (**Figure 4B**). Further supporting the deleterious impact of ERα-MISS abrogation on insulin sensitivity, a defect in Akt Ser473 phosphorylation, which reflects insulin receptor activity following an acute insulin challenge *in vivo*, occurred in the liver of *C451A-ERα* females fed a HFD for 6 weeks (**Figure 4C**). A similar but non-significant trend was observed in skeletal muscle (**Figure 4D**). These results thus provide further evidence that ERα-MISS contributes to the preservation of insulin sensitivity in female mice submitted to a HFD.

### 3.4 Loss of ERα-MISS alters energy expenditure in HFD-fed female mice

**Figure 5:**
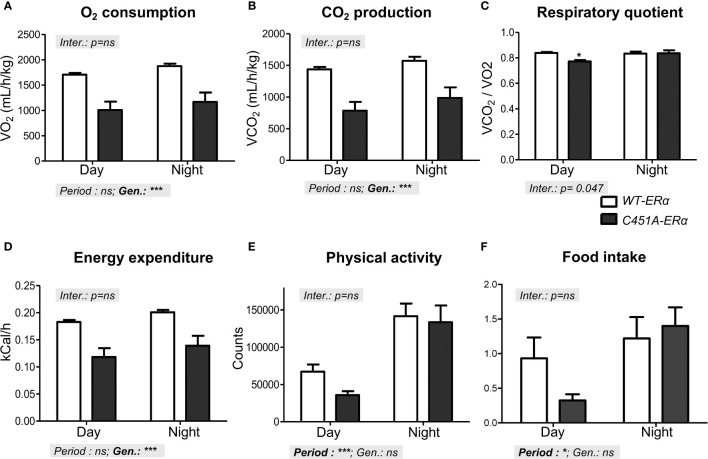
Reduced energy expenditure characterizes HFD-fed *C451A-ERα* female mice Metabolic parameters were registered in metabolic cages in *WT-ERα* and *C451A-ERα* female mice fed a HFD for 4 weeks. Mean values of oxygen consumption **(A)**, CO_2_ production **(B)**, respiratory quotient **(C)**, energy expenditure **(D)**, physical activity **(E)** and food intake **(F)** are shown according to the day (light) and night (dark) periods. Data are shown as mean ± SEM (n= 4/genotype). Two-way ANOVA analyses were used to assess the interaction between the genotype (*WT-ERα versus C451A-ERα*) and the day period (day *versus* night). In the absence of significant interaction, statistical analyses regarding the respective influence of genotype and day period are provided. In case of significant interaction, Bonferroni post-tests were subsequently performed to compare the different groups two by two. Genotype effect during the day period: *, p <0.05.

Since impaired energy expenditure has been proposed as one of the primary causes of obesity in *ERα^-/-^* mice (1, 2), female *C451A-ERα* mice and their *WT-ERα* littermates were then monitored in metabolic cages using indirect calorimetry. Mice were explored after 4 weeks of HFD feeding to avoid significant difference in body weight between the two genotypes (24.8 ± 1.2 *versus* 22.0 ± 0.3 g in *C451A-ERα* and *WT-ERα* mice, respectively). As compared to control mice, O_2_ consumption and CO_2_ production were both reduced in *C451A-ERα* females during the day/passive phase and the night/active phase (**Figures 5A, B**), resulting in a slight decrease in the respiratory quotient only during the day period (**Figure 5C**). Noteworthy, female *C451A-ERα* mice were characterized by a significant decrease in energy expenditure during both day and night periods (**Figure 5D**), without significant concomitant changes in physical activity level (**Figure 5E**) or in food intake (1.7 ± 0.4 *versus* 2.1 ± 0.3 g/kg/day in *C451A-ERα* and *WT-ERα* mice, respectively) (**Figure 5F**). These observations thus indicate that HFD-fed female *C451A-ERα* mice display a decrease in energy expenditure, which could contribute to their exacerbated dysmetabolic phenotype.

### 3.5 Membrane ERα regulates thermogenesis in HFD-fed female mice

**Figure 6:**
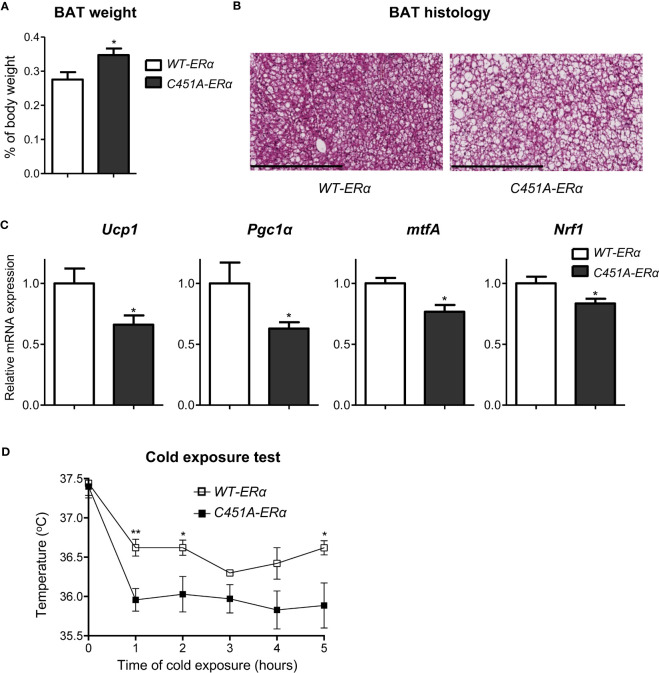
Abolition of membrane ERα signaling alters thermogenesis in HFD-fed *C451A-ERα* female mice. Five-week-old *WT-ERα* and *C451A-ERα* male and female mice were fed a HFD for 12 weeks. Brown adipose tissue (BAT) relative weight **(A)**, representative photomicrograph of BAT sections stained with H/E (scale bar: 100µm) and relative mRNA expression levels of genes involved in thermogenesis and mitochondrial biogenesis **(C)** are shown. qPCR data were normalized to RPL19 mRNA levels. Another set of HFD-fed female mice were submitted to a 5h cold test (4°C). Evolution of body temperature in response to cold exposure is shown **(D)**. Data are shown as mean ± SEM (n= 4-7/genotype). **(A-D)** unpaired Student’s t test was performed. *, genotype effect: *p<0.05, **p<0.01.

To get further insights in the influence of ERα-MISS on energy expenditure regulation, we next decided to explore the thermogenesis status of HFD-fed female *C451A-ERα* mice. As compared to *WT-ERα* mice, mean weight of brown adipose tissue (BAT), a tissue recognized to play a crucial role in thermogenesis processes, was significantly increased in *C451A-ERα* females (**Figure 6A**), and histological analysis revealed a large amount of lipid deposition in BAT adipocytes (**Figure 6B**). Accordingly, mRNA expression levels of key molecular actors of BAT thermogenesis such as *Ucp1* (Uncoupling Protein-1) and *Pgc1α* (Peroxisome-Proliferator-Activated Receptor-gamma Coactivator 1 alpha) were significantly decreased in BAT samples from *C451A-ERα* females (**Figure 6C**). Moreover, mRNA expression levels of *mtfA* (mitochondrial transcription factor A) and *Nrf1* (Nuclear Respiratory Factor-1), both involved in mitochondrial biogenesis and previously reported to be regulated by estrogens (22), were also altered in *C451A-ERα* mice (**Figure 6C**). Such dysfunctional BAT characteristics thus strengthened the hypothesis that altered energy expenditure in HFD-fed *C451A-ERα* female mice could result, at least in part, from an impairment in thermogenesis regulation. Therefore, HFD-fed female *C451A-ERα* mice were submitted to a cold tolerance test that demonstrated their lower ability to adapt their body temperature to cold stress as compared to control females (**Figure 6D**). Taken together, our results thus suggest that ERα-MISS-dependent regulation of BAT thermogenic function could contribute to female protection against HFD-induced metabolic disorders.

## 4 Discussion

The present study highlights the sex-dependent contribution of membrane ERα to the protection conferred by endogenous estrogens against HFD-induced obesity and metabolic disorders. Indeed, abrogation of ERα MISS (selective loss of function) affects female mice, resulting in impaired regulation of energy expenditure and exacerbated features of the metabolic syndrome under nutritional challenge.

The pivotal role of ERα in the regulation of energy balance and metabolic homeostasis was evidenced in both sexes and primarily linked to the classical nuclear activity of the receptor (11). Supporting this assertion, abolition of ERα transcriptional activity in mice deficient for the activation function ERαAF-2 (*ERα AF-2°*) results in fat mass accumulation, insulin resistance and glucose intolerance in both males and females fed either a NCD or a HFD, as observed in *ERα^-/-^* mice (7). Since *ERα AF-2°* mice retains the ability to respond to EDC and thus to mediate ERα MISS effects (20), these data thus suggested a minor, if any, contribution of membrane ERα in estrogen-dependent metabolic protection, at least in the absence of functional nuclear ERα activity (11). Accordingly, the phenotype of the membrane only ERα (*MOER*) mouse model, which only express the ERα E domain at the plasma membrane and are totally devoid of ERα nuclear action, is also very similar to that of *ERα^-/-^* mice, including increased adiposity, insulin resistance and glucose intolerance in both males and females (18, 19, 23).

However, the abolition of ERα MISS effects appears to make females more susceptible to the development of obesity and insulin resistance under deleterious nutritional conditions, modeled by exposure to a HFD in our study. This sex-specific influence of ERα MISS on energy balance and glucose metabolism in HFD-fed animals deserves to be underlined because this sex difference effect contrasts with the complete deletion of the receptor or the abrogation of its transcriptional nuclear effects that affects male as well as female mice, irrespective of the type of diet (7). In mice fed a NCD, no significant change in *C451A-ERα* were found here compared to *WT-ERα* genotype in terms of body weight gain, adiposity and glucose tolerance, up to 7 months of age and regardless of sex. These findings are consistent with the conclusion of experiments conducted in NCD-fed *NOER* mice showing that estrogen-mediated control of body fat mass and liver weight are essentially independent of membrane ERα (24). However, increased visceral fat deposits as well as mildly impaired hepatic insulin sensitivity and glucose tolerance were recently reported in *NOER* females apart from any nutritional challenge (18, 19), suggesting that differences in the experimental settings such as a nutritional stress could reveal the role of membrane ERα in metabolic regulations.

Further characterization of HFD-fed *C451A-ERα* female mice revealed energy balance impairment which can account, at least in part, for their increased susceptibility to metabolic disorders in response to such a nutritional challenge. Indeed, despite similar levels of food intake and physical activity as compared to *WT-ERα* controls, ERα MISS abrogation led to a significant decrease in daily energy expenditure and to impaired thermogenesis capacity, along with BAT histological and functional abnormalities in *C451A-ERα* females. The influence of estrogens on energy expenditure and thermogenesis regulation is well recognized, first demonstrated to be mediated through ERα-dependent actions on the central nervous system (2). More precisely, ERα activation in hypothalamic steroidogenic factor-1 (SF1) neurons has been shown to increase central sympathetic outflow, which consequently enhances BAT thermogenesis and inhibits fat storage in gonadal WAT (25). Although our study did not allow us to conclude whether the absence of membrane ERα specifically impacts the central regulation of energy expenditure, recent data indicate that ERα MISS effects modulate the adaptive capacities of hypothalamic neurons to control energy balance in mice. First, the *C451A-ERα* mutation has been shown to attenuate acute feeding in mice submitted to either fasting or central glucopenia, and to impair the responses of ERα-expressing hypothalamic neurons to hypoglycemia (26). Second, in another knock-in model consisting in the disruption of ERα-striatin binding, which also results in the abolition of ERα MISS actions, female mice were characterized by impaired activation of the protein phosphatase 2A in the hypothalamus, as well as alterations in central modulation of energy balance, adaptive thermogenesis and physical activity (27).

Besides their actions on the central nervous system, estrogens are also able to modulate energy expenditure through direct activation of ERα in peripheral tissues including the BAT. Accordingly, experiments using mice with a selective deletion of *Esr1* from brown adipocytes recently showed that ERα signaling in BAT adipocytes is absolutely required for both mitochondrial remodeling by dynamin-related protein 1 (*Drp1*) and uncoupled respiration thermogenesis by *Ucp1* (28). Moreover, *MOER* mice seem to be more sensitive than *NOER* mice to the administration of a beta-adrenergic receptor agonist, suggesting a facilitating role for ERα MISS on lipolysis and beiging induction in white adipocytes (29). Complementary roles of membrane and nuclear ERα were also demonstrated in: i) the inhibitory action of E2 on insulin-stimulated triglyceride synthesis and storage by cultured bone marrow-derived mature adipocytes (18), and ii) the inhibitory action of E2 on the rosiglitazone-induced differentiation of 3T3-L1 pre-adipocytes and adipose-derived stem cells (ADSC) to mature adipocytes (30). Thus, ERα MISS enhances energy expenditure in HFD-fed females probably through combined actions on central and peripheral tissue targets, whose respective roles remain to be definitely established.

Otherwise, the protection of female mice against HFD-induced insulin resistance could involve ERα MISS effects in other tissues involved in glucose and lipid metabolism, and more specifically in the liver. Supporting this assumption, *NOER* female mice have been reported to exhibit mild hepatic insulin resistance (19). Accordingly, we observed a defect in insulin receptor pathway activation following acute insulin administration *in vivo*, in liver samples from *C451A-ERα* female mice fed a HFD for only 4 weeks. Moreover, our results showing a marked increase in liver triglycerides in HFD-fed *C451A-ERα* females are in perfect agreement with these previous observations. In fact, activation of ERα MISS alone appeared sufficient to decrease insulin-stimulated lipogenesis in hepatocytes of *MOER* mice (31) and selective activation of ERα MISS by EDC prevented hepatic steatosis in female mice (15). Finally, the impaired glucose tolerance observed in HFD-fed *C451A-ERα* females could have involved ERα MISS effects on pancreatic β-cell function. Indeed, membrane ERα was reported to potentiate glucose-stimulated insulin biosynthesis through kinase activation promoting insulin gene transcription (16), but also to promote both mouse and human islet survival in response to pro-apoptotic challenges *in vitro* (32, 33).

The present data reinforce the evidence that membrane and nuclear ERα collaborate to confer optimal protection of female mice against metabolic disorders. In the past decade, combination of genetic and pharmacological approaches revealed that membrane and nuclear ERα actions could either predominate or closely interact according to the cell/tissue and function studied (10, 13). For instance, some vascular actions of estrogens (nitric oxide (NO)-dependent vasorelaxation or acceleration of endothelial healing) appear to be exclusively dependent on ERα MISS, while others (atheroma prevention and artery remodeling) are mainly dependent on ERα nuclear signaling (20, 34, 35). In contrast, close interaction between membrane and nuclear ERα was reported in bones (24), and probably occurs in tissues controlling energy balance and carbohydrate metabolism, depending on cell types but also on the nutritional conditions. In this paradigm, rapid activation of extra-nuclear signaling pathways by membrane ERα probably contributes to potentiate the downstream nuclear translocation and the transcriptional activity of the receptor (10, 13), as shown for the actions of E2 on both mature adipocyte differentiation and insulin synthesis (16, 30).

Finally, some experimental considerations deserve to be discussed to properly interpret the present data, especially regarding the initiation and the duration of the HFD feeding. First, to be able to analyze the respective influence of nuclear and membrane ERα signaling under standardized conditions, mice were switched to HFD from 5 weeks of age, as used in our previous study evaluating the contribution of ERα activation functions to the metabolic effects of estrogens (7). Nevertheless, we acknowledge that the age at HFD feeding initiation is likely to influence the course of obesity and associated metabolic disorders in a sex-specific manner (36, 37), although conflicting studies have been reported (38, 39). Second, in mice fed a HFD for 12 weeks, the more severe obese phenotype of *C451A-ERα* females as compared to their *WT* littermates could have contributed to impairments in insulin sensitivity or BAT function. Although we cannot rule out this hypothesis, this is the reason why some experiments were carried out with shorter periods of HFD exposure, demonstrating altered energy expenditure and impaired insulin signaling in *C451A-ERα* female mice after only 4 or 6 weeks of HFD feeding, respectively. Thus, further studies are now required to identify and prioritize the molecular mechanisms contributing to female metabolic protection under the control of the membrane receptor in HFD-fed mice. For instance, besides the description of WAT hypertrophy in HFD-fed *C451A-ERα* female mice, characterized by the appearance of large adipocytes, whether the alteration of membrane ERα signaling promotes the development of new large-sized adipocytes and/or enhances lipid storage in pre-existing adipocytes remains to be explored.

In summary, the present study provides additional evidence that membrane ERα contributes to the beneficial metabolic actions of estrogens, highlighting the crucial role of ERα MISS effects in female protection against the adverse effects of an obesogenic diet. The interactions between membrane and nuclear ERα separate actions and cross-talks are undoubtedly highly complex, as a consequence of the influence of tissue specificities, time course and dose of estrogen exposure, nutritional status or other environmental factors, etc. Regardless of such a level of complexity in molecular interactions, our data further support the potential clinical implications of pathway-selective membrane ERα modulation to enhance energy expenditure and to preserve insulin sensitivity in clinical situations of increased risk for obesity-related metabolic disorders such as T2D or NAFLD.

## Data availability statement

The original contributions presented in the study are included in the article/**Supplementary Material**. Further inquiries can be directed to the corresponding author.

## Ethics statement

All experimental procedures were performed in accordance with the principles established by EU directive 2010/63/EU, the Institut National de la Santé et de la Recherche Médicale and approved by the local Ethical Committee of Animal Care (CEA-122-DAP-2014-56).

## Author contributions

AF, J-FA and PG conceived and designed the study. AF, BT, AM, CM, ER, MA, CF, FL contributed to the provision of the *C451A-ERα* mouse model and/or to the carrying out of experimental procedures. RB supervised hyperinsulinemic-euglycemic clamps. AF, BT, AM, M-LC and PG analyzed and interpreted the results. AF, BT, AM and PG wrote the manuscript. All authors reviewed the manuscript. All authors contributed to the article and approved the submitted version.

## Supplementary material

Supplementary Figure 1 Intraperitoneal glucose tolerance test in HFD-fed *C451A-ERα* female mice fed a HFD for 4 and 8 weeks, respectively. Five-week-old *WT-ERα* and *C451A-ERα* female mice were fed a HFD for 3 months. Intraperitoneal glucose tolerance performed after 1 **(A)** and 2 **(B)** months of HFD feeding are shown. Data are shown as mean ± SEM (n= 4-6/genotype). Repeated measures ANOVA were used to compare changes over time between the two genotypes. For AUC, Unpaired Student’s t test was performed. *, genotype effect: *p<0.05, **p<0.01 and ***p<0.001.

Supplementary Figure 2 Food intake in NCD or HFD-fed *WT-ERα* and *C451A-ERα* male and female mice. Five-week-old *WT-ERα* and *C451A-ERα* male and female mice were maintained on a normal chow diet (NCD) up to 7 months of age or fed a high-fat diet (HFD) for 3 months. Food intakes in NCD **(A, B)** and in HFD **(C, D)** groups are shown in male and female mice, respectively. Data are shown as mean ± SEM (n= 4-15/genotype/sex). **(A-D)** Unpaired Student’s t test was performed. *, genotype effect: ***p<0.001.

## References

[1] Mauvais-Jarvis F Clegg DJ Hevener AL. The role of estrogens in control of energy balance and glucose homeostasis. Endocr Rev (2013) 34:309–38. doi: 10.1210/er.2012-1055 PMC3660717 23460719

[2] Morselli E Santos RS Criollo A Nelson MD Palmer BF Clegg DJ. The effects of oestrogens and their receptors on cardiometabolic health. Nat Rev Endocrinol (2017) 13:352–64. doi: 10.1038/nrendo.2017.12 28304393

[3] Tramunt B Smati S Grandgeorge N Lenfant F Arnal J-F Montagner A. Sex differences in metabolic regulation and diabetes susceptibility. Diabetologia (2020) 63:453–61. doi: 10.1007/s00125-019-05040-3 PMC6997275 31754750

[4] Hewitt SC Korach KS. Estrogen receptors: new directions in the new millennium. Endocr Rev (2018) 39:664–75. doi: 10.1210/er.2018-00087 PMC6173474 29901737

[5] Heine PA Taylor JA Iwamoto GA Lubahn DB Cooke PS. Increased adipose tissue in male and female estrogen receptor-alpha knockout mice. Proc Natl Acad Sci USA (2000) 97:12729–34. doi: 10.1073/pnas.97.23.12729 PMC18832 11070086

[6] Cooke PS Heine PA Taylor JA Lubahn DB. The role of estrogen and estrogen receptor-alpha in male adipose tissue. Mol Cell Endocrinol (2001) 178:147–54. doi: 10.1016/S0303-7207(01)00414-2 11403904

[7] Handgraaf S Riant E Fabre A Waget A Burcelin R Lière P. Prevention of obesity and insulin resistance by estrogens requires ERα activation function-2 (ERαAF-2), whereas ERαAF-1 is dispensable. Diabetes (2013) 62:4098–108. doi: 10.2337/db13-0282 PMC3837069 23903353

[8] Smith EP Boyd J Frank GR Takahashi H Cohen RM Specker B. Estrogen resistance caused by a mutation in the estrogen-receptor gene in a man. N Engl J Med (1994) 331:1056–61. doi: 10.1056/NEJM199410203311604 8090165

[9] Riant E Waget A Cogo H Arnal J-F Burcelin R Gourdy P. Estrogens protect against high-fat diet-induced insulin resistance and glucose intolerance in mice. Endocrinology (2009) 150:2109–17. doi: 10.1210/en.2008-0971 19164473

[10] Arnal J-F Lenfant F Metivier R Flouriot G Henrion D Adlanmerini M. Membrane and nuclear estrogen receptor alpha actions: from tissue specificity to medical implications. Physiol Rev (2017) 97:1045–87. doi: 10.1152/physrev.00024.2016 28539435

[11] Gourdy P Guillaume M Fontaine C Adlanmerini M Montagner A Laurell H. Estrogen receptor subcellular localization and cardiometabolism. Mol Metab (2018) 15:56–69. doi: 10.1016/j.molmet.2018.05.009 29807870 PMC6066739

[12] Adlanmerini M Fontaine C Gourdy P Arnal J-F Lenfant F. Segregation of nuclear and membrane-initiated actions of estrogen receptor using genetically modified animals and pharmacological tools. Mol Cell Endocrinol (2022) 539:111467. doi: 10.1016/j.mce.2021.111467 34626731

[13] Mauvais-Jarvis F Lange CA Levin ER. Membrane-initiated estrogen, androgen, and progesterone receptor signaling in health and disease. Endocr Rev (2022) 43:720–42. doi: 10.1210/endrev/bnab041 PMC9277649 34791092

[14] Madak-Erdogan Z Kim SH Gong P Zhao YC Zhang H Chambliss KL. Design of pathway preferential estrogens that provide beneficial metabolic and vascular effects without stimulating reproductive tissues. Sci Signal (2016) 9:ra53. doi: 10.1126/scisignal.aad8170 27221711 PMC4896643

[15] Chambliss KL Barrera J Umetani M Umetani J Kim SH Madak-Erdogan Z. Nonnuclear estrogen receptor activation improves hepatic steatosis in female mice. Endocrinology (2016) 157:3731–41. doi: 10.1210/en.2015-1629 PMC5045504 27552247

[16] Wong WPS Tiano JP Liu S Hewitt SC Le May C Dalle S. Extranuclear estrogen receptor-alpha stimulates NeuroD1 binding to the insulin promoter and favors insulin synthesis. Proc Natl Acad Sci U.S.A. (2010) 107:13057–62. doi: 10.1073/pnas.0914501107 PMC2919966 20616010

[17] Tiano JP Delghingaro-Augusto V Le May C Liu S Kaw MK Khuder SS. Estrogen receptor activation reduces lipid synthesis in pancreatic islets and prevents β cell failure in rodent models of type 2 diabetes. J Clin Invest (2011) 121:3331–42. doi: 10.1172/JCI44564 PMC3148728 21747171

[18] Pedram A Razandi M Blumberg B Levin ER. Membrane and nuclear estrogen receptor α collaborate to suppress adipogenesis but not triglyceride content. FASEB J (2016) 30:230–40. doi: 10.1096/fj.15-274878 PMC4684544 26373802

[19] Allard C Morford JJ Xu B Salwen B Xu W Desmoulins L. Loss of nuclear and membrane estrogen receptor-α differentially impairs insulin secretion and action in Male and female mice. Diabetes (2019) 68:490–501. doi: 10.2337/db18-0293 30305367 PMC6385757

[20] Adlanmerini M Solinhac R Abot A Fabre A Raymond-Letron I Guihot A-L. Mutation of the palmitoylation site of estrogen receptor α *in vivo* reveals tissue-specific roles for membrane versus nuclear actions. Proc Natl Acad Sci U.S.A. (2014) 111:E283–290. doi: 10.1073/pnas.1322057111 PMC3896153 24371309

[21] Nilsson ME Vandenput L Tivesten Å Norlén A-K Lagerquist MK Windahl SH. Measurement of a comprehensive sex steroid profile in rodent serum by high-sensitive gas chromatography-tandem mass spectrometry. Endocrinology (2015) 156:2492–502. doi: 10.1210/en.2014-1890 25856427

[22] Klinge CM. Estrogenic control of mitochondrial function and biogenesis. J Cell Biochem (2008) 105:1342–51. doi: 10.1002/jcb.21936 PMC2593138 18846505

[23] Pedram A Razandi M Kim JK O’Mahony F Lee EY Luderer U. Developmental phenotype of a membrane only estrogen receptor alpha (MOER) mouse. J Biol Chem (2009) 284:3488–95. doi: 10.1074/jbc.M806249200 PMC2635032 19054762

[24] Gustafsson KL Farman H Henning P Lionikaite V Movérare-Skrtic S Wu J. The role of membrane ERα signaling in bone and other major estrogen responsive tissues. Sci Rep (2016) 6:29473. doi: 10.1038/srep29473 27388455 PMC4937452

[25] Xu Y Nedungadi TP Zhu L Sobhani N Irani BG Davis KE. Distinct hypothalamic neurons mediate estrogenic effects on energy homeostasis and reproduction. Cell Metab (2011) 14:453–65. doi: 10.1016/j.cmet.2011.08.009 PMC3235745 21982706

[26] Yu K He Y Hyseni I Pei Z Yang Y Xu P. 17β-estradiol promotes acute refeeding in hungry mice via membrane-initiated ERα signaling. Mol Metab (2020) 42:101053. doi: 10.1016/j.molmet.2020.101053 32712433 PMC7484552

[27] Ueda K Takimoto E Lu Q Liu P Fukuma N Adachi Y. Membrane-initiated estrogen receptor signaling mediates metabolic homeostasis *via* central activation of protein phosphatase 2A. Diabetes (2018) 67:1524–37. doi: 10.2337/db17-1342 PMC6054435 29764860

[28] Zhou Z Moore TM Drew BG Ribas V Wanagat J Civelek M. Estrogen receptor α controls metabolism in white and brown adipocytes by regulating Polg1 and mitochondrial remodeling. Sci Transl Med (2020) 12:eaax8096. doi: 10.1126/scitranslmed.aax8096 32759275 PMC8212422

[29] Santos RS Frank AP Fátima LA Palmer BF Öz OK Clegg DJ. Activation of estrogen receptor alpha induces beiging of adipocytes. Mol Metab (2018) 18:51–9. doi: 10.1016/j.molmet.2018.09.002 PMC6309577 30270132

[30] Ahluwalia A Hoa N Ge L Blumberg B Levin ER. Mechanisms by which membrane and nuclear ER alpha inhibit adipogenesis in cells isolated from female mice. Endocrinology (2020) 161:bqaa175. doi: 10.1210/endocr/bqaa175 32976570

[31] Pedram A Razandi M O’Mahony F Harvey H Harvey BJ Levin ER. Estrogen reduces lipid content in the liver exclusively from membrane receptor signaling. Sci Signal (2013) 6:ra36. doi: 10.1126/scisignal.2004013 23695162

[32] Liu S Le May C Wong WPS Ward RD Clegg DJ Marcelli M. Importance of extranuclear estrogen receptor-alpha and membrane G protein-coupled estrogen receptor in pancreatic islet survival. Diabetes (2009) 58:2292–302. doi: 10.2337/db09-0257 PMC2750222 19587358

[33] Tiano JP Mauvais-Jarvis F. Importance of oestrogen receptors to preserve functional β-cell mass in diabetes. Nat Rev Endocrinol (2012) 8:342–51. doi: 10.1038/nrendo.2011.242 22330739

[34] Chambliss KL Wu Q Oltmann S Konaniah ES Umetani M Korach KS. Non-nuclear estrogen receptor alpha signaling promotes cardiovascular protection but not uterine or breast cancer growth in mice. J Clin Invest (2010) 120:2319–30. doi: 10.1172/JCI38291 PMC2898582 20577047

[35] Guivarc’h E Buscato M Guihot A-L Favre J Vessières E Grimaud L. Predominant role of nuclear versus membrane estrogen receptor α in arterial protection: implications for estrogen receptor α modulation in cardiovascular Prevention/Safety. J Am Heart Assoc (2018) 7:e008950. doi: 10.1161/JAHA.118.008950 29959137 PMC6064913

[36] Nishikawa S Yasoshima A Doi K Nakayama H Uetsuka K. Involvement of sex, strain and age factors in high fat diet-induced obesity in C57BL/6J and BALB/cA mice. Exp Anim (2007) 56:263–72. doi: 10.1538/expanim.56.263 17660680

[37] Glavas MM Lee AY Miao I Yang F Mojibian M O’Dwyer SM. Developmental timing of high-fat diet exposure impacts glucose homeostasis in mice in a sex-specific manner. Diabetes (2021) 70:2771–84. doi: 10.2337/db21-0310 PMC8660987 34544729

[38] Salinero AE Anderson BM Zuloaga KL. Sex differences in the metabolic effects of diet-induced obesity vary by age of onset. Int J Obes (Lond) (2018) 42:1088–91. doi: 10.1038/s41366-018-0023-3 29463918

[39] Cordoba-Chacon J Gahete MD Pozo-Salas AI Moreno-Herrera A Castaño JP Kineman RD. Peripubertal-onset but not adult-onset obesity increases IGF-I and drives development of lean mass, which may lessen the metabolic impairment in adult obesity. Am J Physiol Endocrinol Metab (2012) 303:E1151–1157. doi: 10.1152/ajpendo.00340.2012 PMC3774069 22932784

