# Supplementary material for: Membrane estrogen receptor-α contributes to female protection against high-fat diet-induced metabolic disorders
Source: Front Endocrinol (Lausanne). 2023 Jul 17;14:1215947. doi: 10.3389/fendo.2023.1215947 (PMC10390233; doi:10.3389/fendo.2023.1215947)
Supplement: Supplementary Figure 1 — Intraperitoneal glucose tolerance test in HFD-fed C451A-ERα female mice fed a HFD for 4 and 8 weeks, respectively. Five-week-old WT-ERα and C451A-ERα female mice were fed a HFD for 3 months. Intraperitoneal glucose tolerance performed after 1 (A) and 2 (B) months of HFD feeding are shown. Data are shown as mean ± SEM (n= 4-6/genotype). Repeated measures ANOVA were used to compare changes over time between the two genotypes. For AUC, Unpaired Student’s t test was performed. *, genotype effect: *p<0.05, **p<0.01 and ***p<0.001. [file DataSheet_1.pdf]

Supplementary Figure 1

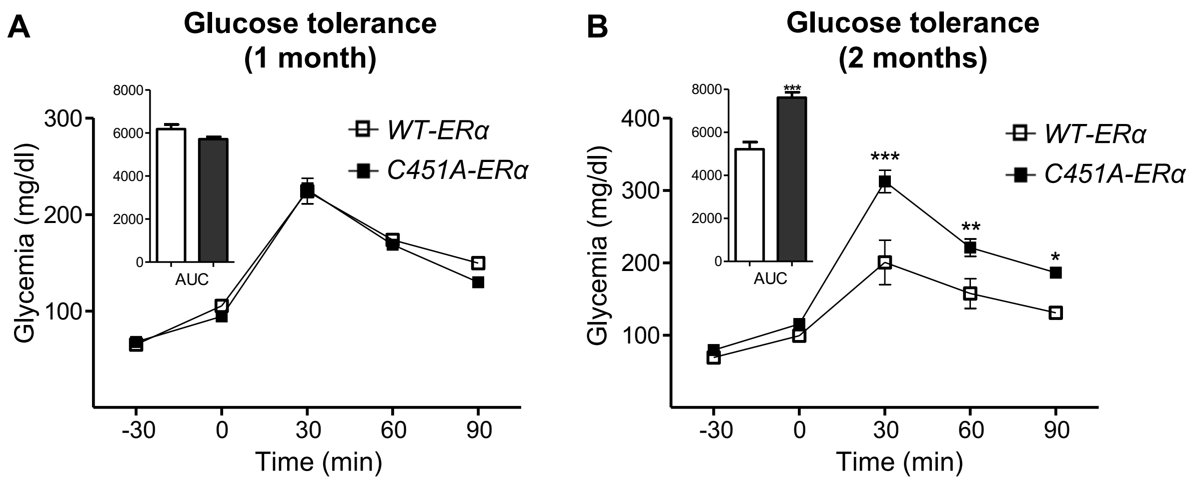

Supplementary Figure 2

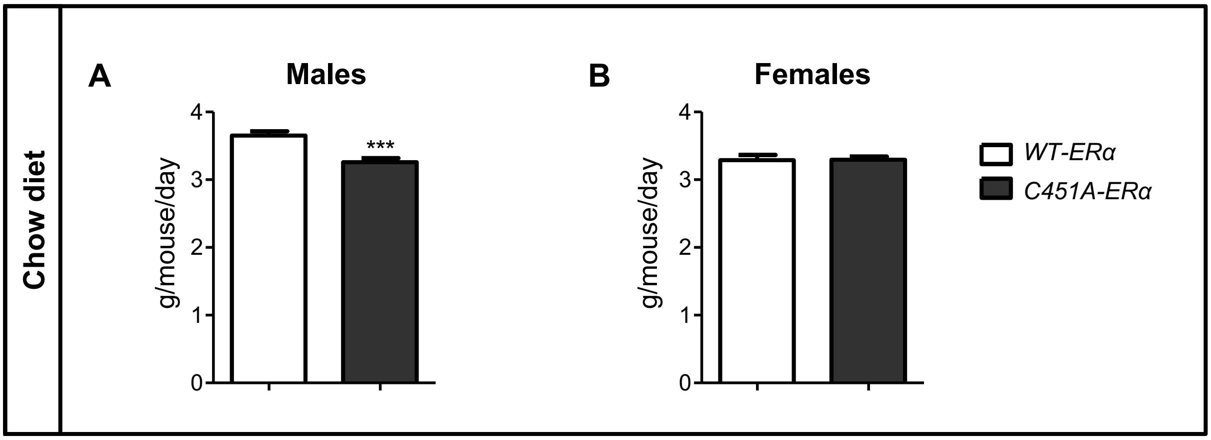

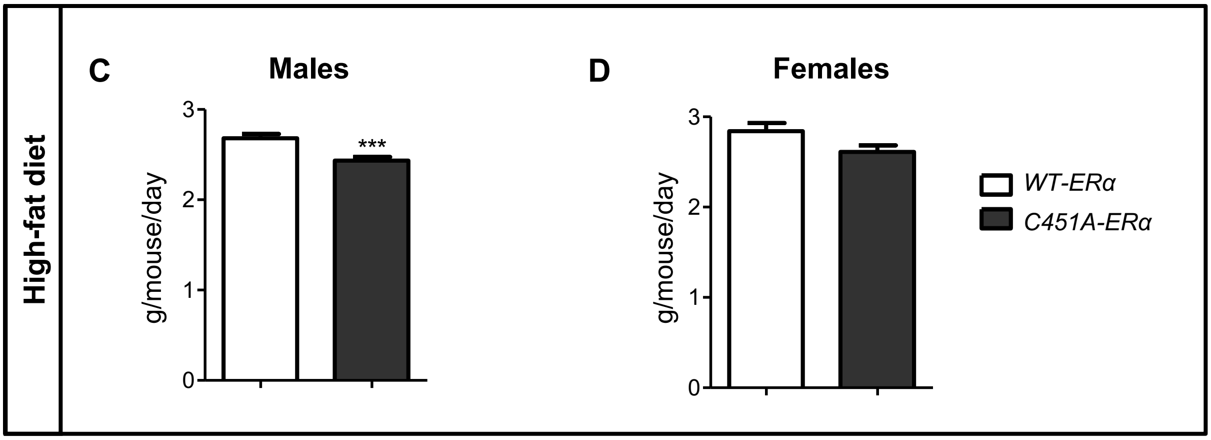

Supplementary Table 1. Primers sequences

Ucp1
F : CCTGCCTCTCTCGGAAACAA
R : TGTAGGCTGCCCAATGAACA
NM_009463

Glut4
F: CCGGATTCCATCCCACAAG
R: CATGCCACCCACAGAGAAGA
NM_009204

mtfA
F: GGAGGCAAAGGATGATTCGGC
R: AGCTGAATATATGCCTGCTTTTCCT
NM_009360

Nrf1
F: TAACTGCCCATCTGGCTGCTG
R: TCTGAGTTTCCGAAGCAAACGTG
NM_001410232

PGC1α
F : TCACGTTCAAGGTCACCCTA
R : TCTCTCTCTGTTTGGCCCTT
NM_008904

Ppia
F : GAGCTGTTTGCAGACAAAGTTC
R : CCCTGGCACATGAATCCTGG
NM_008907

Rpl19
F: TGCGGCCCACAAGCTCTTTCC
R: TTCCCGCAGCGGAGGACACT
NM_009078

HPRT
F: GCCAGACTTTGTTGGATTTGAA
R: CGCTCATCTTAGGCTTTGTATTTG
NM_013556

36B4
F: ACTTCTCTTAAAACTCCGGTCTGG
R: GCGGTTTTGCTTTTTCATCCTGC
NM_007475

Supplementary Table 2. Concentrations of circulating factors in HFD-fed *WT-ERα* and *C451A-ERα* female mice.

	WT-ERα	*C451A-ERα*	p value (t-test)
**Sex steroids**			
Estradiol (pg/mL)	24.6 ± 12.7	24.8 ± 11	0.98
Testosterone (pg/mL)	22.9 ± 9.4	76.5 ± 21.1	0.06
Progesterone (pg/mL)	2680 ± 1530	1463 ± 486	**0.05**
Androstenedione (pg/mL)	54.7 ± 9.5	77.4 ± 16.3	0.27
**Lipid profile**			
Total cholesterol (mmol/L)	1.92 ± 0.24	2.65 ± 0.09	**0.01**
HDL cholesterol (mmol/L)	1.03 ± 0.16	1.39 ± 0.03	**0.04**
Triglycerides (mmol/L)	0.48 ± 0.09	0.45 ± 0.02	0.77
**Adipokines**			
Leptin (ng/mL)	4.47 ± 1.71	21.61 ± 1.29	**< 0.0001**
Resistin (ng/mL)	2.14 ± 0.09	3.09 ± 0.32	**0.04**
Adiponectin (ng/mL)	21050 ± 1784	22120 ± 979.3	0.59
**Transaminases**			
ASAT (UI/L)	24.6 ± 12.7	24.8 ± 11	0.98
ALAT (UI/L)	22.9 ± 9.4	76.5 ± 21.1	0.06
